# Unconscious processing of happy faces correlates with prosocial tendency but not extraversion

**DOI:** 10.3389/fpsyg.2024.1458373

**Published:** 2025-02-04

**Authors:** Qian Xu

**Affiliations:** ^1^Department of Psychology, Beijing Union University, Beijing, China; ^2^Learning and Psychological Development Institution for Children and Adolescents, Beijing Union University, Beijing, China

**Keywords:** unconscious, emotional face, happy face, prosocial tendency, extraversion

## Abstract

Perceiving facial expressions plays a crucial role in face-to-face social interactions. A wealth of studies has revealed the unconscious processing of emotional stimuli, including facial expressions. However, the relationship between the unconscious processing of happy faces and socially oriented personality traits—such as extraversion and prosocial tendency—remains largely unexplored. By pairing backward-masked faces with supraliminally presented faces in both visual fields, we found that the discrimination of visible emotional faces was modulated by the facial expressions of the invisible faces in the opposite visual field. The emotionally consistent condition showed a shorter reaction time (Exp 1) or higher accuracy (Exp 2) than the inconsistent condition. Moreover, the unconscious processing of happy faces was positively correlated with prosocial tendency but not with extraversion personality. These findings shed new light on the adaptive functions of unconscious emotional face processing, and highlight the importance of future investigations into the unconscious processing of extrafoveal happy expression.

## 1 Introduction

Facial expressions play a crucial role in social communication (Tracy et al., 2015). Happy expressions can convey trust, friendliness, approval, or liking. Angry expressions might signal hostility or dissatisfaction, while fearful expressions can suggest vigilance or potential danger. Our perception of others’ facial expressions often influences our subsequent decisions and behaviors toward them.

Over the past three decades, numerous studies have shown that emotional stimuli, such as facial expressions, can be registered by parts of the brain, including the amygdala, even when they are not consciously perceived (Bertini and Làdavas, 2021; Diano et al., 2016; Jiang and He, 2006; Pegna et al., 2005; Qiu et al., 2022; Tamietto and De Gelder, 2010; Wang et al., 2023). This unconscious perception can influence social perception (Anderson et al., 2012; Gruber et al., 2016; Sagliano et al., 2020), and guide behaviors (Almeida et al., 2013) or eye movements (Vetter et al., 2019). “Unconscious” refers to sensory input below the consciousness threshold in psychophysics. Only a small percentage of sensory input triggers conscious perception. There are two types of unconsciousness (or unawareness): sensory and attentional (Tamietto and De Gelder, 2010). Sensory unconsciousness occurs when stimuli are presented with extremely weak strength, such as being too short in duration or too low in contrast, leading us to perceive nothing even when we are actively paying attention to the stimuli (Tamietto and De Gelder, 2010). Through a forced-choice awareness check procedure conducted after the main experiment, researchers could measure the objective awareness level. Some studies employed a trial-by-trial awareness check procedure to rigorously ensure sensory unconsciousness (Liu et al., 2023; Skora et al., 2024).

Previous research has suggested significant individual differences in the unconscious processing of facial expressions. For instance, a series of studies (Alkozei and Killgore, 2015; Cui et al., 2014; Günther et al., 2020; Günther et al., 2023; Vizueta et al., 2012) demonstrated correlations between the neuroimaging response to the subliminal fearful faces and psychopathology-related traits, such as negative affectivity which entails anxiety, depression, neuroticism, and so on (Günther et al., 2020; Vizueta et al., 2012). However, little is known about the individual differences in the unconscious processing of happy faces, particularly in extrafoveal vision.

Behavioral-level experimental studies have developed methods for measuring the degree of unconscious perception of extrafoveal emotional faces. Research by Bertini et al. (2013) and Tamietto and Gelder (2008) presented participants with visible and visually masked emotional faces (happy and fearful) in both visual fields, respectively, asking them to make emotional judgments about the visible face. They consistently found that when the invisible and visible emotional faces had the same expression, reaction times were significantly shorter than in other conditions. This “interhemispheric interaction between seen and unseen facial expressions” (Tamietto and Gelder, 2008) provides a behavioral measure for the unconscious processing of emotional faces, particularly in extrafoveal vision.

Since facial expressions convey crucial social communicative information (Frith, 2009), we hypothesize that unconscious processing of facial expressions might enhance social interactions. We predict that the stronger one’s ability to unconsciously perceive others’ facial expressions, the stronger their social communication abilities, and consequently, the more pronounced their extroverted personality. In addition, the happy facial expression is the most recognizable facial expression, which is a positive sign of prosocial intentions that is recognized in even the most remote cultures (Ekman and Friesen, 1971). Happy faces were identified most accurately and quickly compared to other facial expressions, even in the extrafoveal visual field (Calvo et al., 2010). Therefore, we also hypothesize that unconsciously perceiving happiness in others’ faces might facilitate our prosocial behaviors toward them. In other words, unconscious processing of happy facial expressions might enhance one’s prosocial tendency. It’s important to distinguish between extraversion and prosocial tendency, as they are distinct and unrelated personality traits (Kline et al., 2019). Extraversion refers to an individual’s enjoyment of and tendency to seek out social interactions. In contrast, prosocial tendency describes one’s inclination to act in ways that benefit others during social interactions. Notably, introverted individuals can also display high levels of prosocial behavior. Thus, these two personality traits—introversion and prosocial tendency—are separate and not necessarily correlated.

The present study aims to investigate whether there is a significant positive correlation between the unconscious processing of happy faces and socially oriented personality traits, particularly extraversion and prosocial tendency. The unconscious processing of happy faces was measured at the behavioral level, using the paradigm of previous studies (Bertini et al., 2013; Tamietto and Gelder, 2008). Experiment 1 investigates the unconscious processing of happy faces. Experiment 2 further validates the phenomenon and explores its correlation with prosocial tendency and extraversion personality traits.

## 2 Experiment 1: unconscious processing of emotional faces

### 2.1 Participants

A repeated-measures, within-factors power analysis in G*Power indicated a minimum sample size of 14 to achieve appropriate power to detect a medium effect size (parameters: effect size *f*(U) = 0.5, α = 0.05, power = 0.85, number of groups = 1, number of measurements = 6, “as in SPSS” option enabled) (Faul et al., 2009).

A total of 27 undergraduates participated in this study. Nine participants were excluded due to high performances in the post-experiment awareness check procedure (see section “2.5.1 Exclusion criteria”). Therefore, there were 18 valid participants (4 males and 14 females), with an average age of 19.40 ± 4.98 years. All participants reported normal or corrected-to-normal visual acuity and were naive to the purpose of the experiment. They all gave written informed consent in accordance with procedures and protocols approved by the institutional review board of our university and received payment for their participation.

### 2.2 Materials

#### 2.2.1 Apparatus and display

The experiment was conducted in an independent cubicle laboratory. Stimuli were displayed on a Tsinghua Tongfang CRT display with a refresh rate of 60 Hz (resolution 1280 × 1024 pixels). The participants were seated in a chair with a chin rest fixed 57 cm from the monitor. The experimental program was written in MATLAB 7.1 (MathWorks) using the Psychtoolbox-3 platform (Brainard, 1997; Kleiner et al., 2007; Pelli, 1997).

#### 2.2.2 Stimuli

**FIGURE 1:**
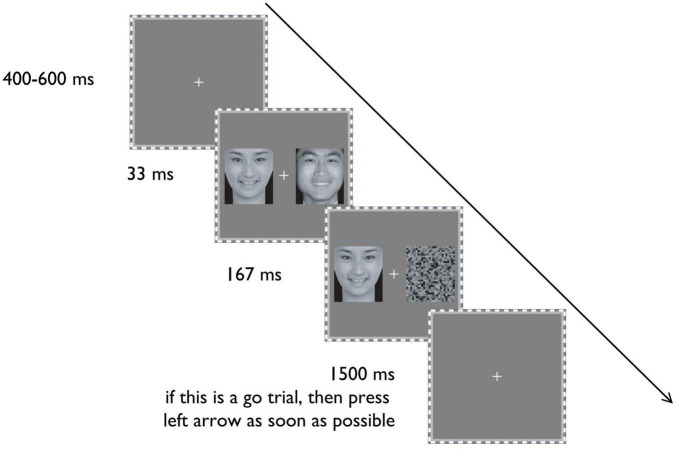
The schematic display of the procedure of a typical trial. Reproduced with permission from Gong et al. (2011).

From the Chinese Emotional Face System (Gong et al., 2011), we selected pictures of happy, fearful, and neutral faces. Six happy and six fearful faces, with high degrees of arousal, were selected, each of which had 3 male and female faces. In addition, six neutral faces (3 male and 3 female) were chosen as the invisible neutral faces in the experiment. Another 180 new neutral faces (half male and half female) were selected and spatially scrambled, serving as masks (see Figure 1).

The mean valence of the six fearful faces (2.79) is smaller than that of the neutral faces (5.00), which is smaller than that of the happy faces (6.58). The differences between either two types of faces reached statistically significant levels (*p*s < 0.003).

The mean arousal of the six fearful faces (5.89) is approximately equal to that of happy faces (5.78), which is higher than that of neutral faces (3.33). There is no significant difference between the arousals of fearful and happy faces (*p* ≈ 1.0), but both are significantly higher than the arousals of neutral faces (*p*s < 0.001).

Finally, we adjusted the average RGB luminance of each face to around 128 through Photoshop software.

### 2.3 Procedure

As shown in Figure 1, each trial starts with a fixation at the center of the screen for 400–600 ms. Subsequently, an emotional (either happy or fearful) face appears on one side of the screen (randomly left or right side) for 200 ms. Simultaneously, on the opposite side, an emotional (either happy or fearful) or neutral face is displayed for 33 ms, followed by a spatially scrambled neutral face for 167 ms as a backward mask.

The experimental task is the classical Go/no-go task (Tamietto and Gelder, 2008). Participants must press the left arrow key on the keyboard as quickly as possible when they detect the target facial expression (Go response). They should not respond when they detect the non-target expression (No-go response). The target expression of the Go reaction is fixed in each block. The target face is the 200-ms visible emotional (either happy or fearful) face. Each trial has a time limit of 1.5 s. The next trial begins after 1.5 s or a key response.

Before the formal experiment, the participants practice until they are familiar with the task. The formal experiment consists of 4 blocks, each with 90 trials. The target expression of the Go reaction is fixed within each block. For half the participants, the target expressions for the four blocks are happy, fear, fear, and then happy. For the other half, the sequence is fear, happy, happy, and then fear. These two ABBA sequences are balanced across participants.

At the end of the experiment, the participants complete an awareness check task. This task follows the same procedure as the formal experiment, but with half the number of trials and a modified experimental task. Participants must choose or guess whether the briefly presented face before the mask (that is, the 167-ms scrambled neutral faces) is emotional or neutral.

### 2.4 Experimental design

The experiment employs a 2 (emotion of the visible face: happy or fearful) × 3 (emotional consistency between visible and invisible faces: consistent, irrelevant, or inconsistent) within-subject design. There are six experimental conditions. Each condition has 60 trials—30 Go trials and 30 No-go trials.

### 2.5 Results and discussions

#### 2.5.1 Exclusion criteria

We calculated the mean and standard deviation of 27 participants on these indices: the overall accuracy and reaction time, and the *d*’ of the awareness check task, which is calculated by the signal detection theory method, with emotional faces as signals and neutral faces as noises.

Those who exceeded the mean ± 2.5 standard deviation of all participants on any of the above indices were excluded. Finally, 9 participants’ *d*’ excluded the mean + 2.5SD (standard deviation) of all participants in the awareness check task.

Therefore, 18 participants were included in the final analysis. Their mean accuracy of Go trials was 0.94, and the mean false alarm rate of No-go Trials was 0.12.

#### 2.5.2 Awareness check results

In the final awareness check task, the mean *d*’ of all participants was 0.08, which was not significantly different from 0 (*t*(17) = 1.629, *p* = 0.122). This demonstrated that the participants could not distinguish whether the 33-ms masked face was emotional or neutral.

#### 2.5.3 Unconscious processing of emotional faces

**FIGURE 2:**
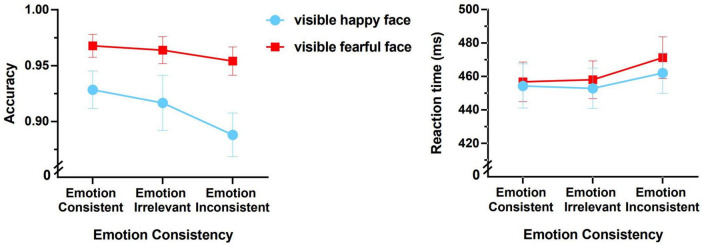
Accuracies and reaction times of the Go/no-go task (the error bars denote ± 1 SEM).

The mean accuracies and reaction times for the six experimental conditions are shown in Figure 2.

*Accuracy*

A 2 (emotion of the visible face: happy or fearful) × 3 (emotional consistency between visible and invisible faces: consistent, irrelevant, or inconsistent) repeated-measures ANOVA of accuracy only revealed a significant main effect of “emotion of the visible face” (*F*(1,17) = 12.782, *p* = 0.002, η_*p*_^2^ = 0.429; the left panel of Figure 2). The fearful face condition shows significantly higher accuracy than the happy face condition. The main effect of emotional consistency was marginally significant (*F*(2,34) = 3.012, *p* = 0.062, η_*p*_^2^ = 0.151). The interaction between them was not significant (*F*(2,34) = 0.627, *p* = 0.540, η_*p*_^2^ = 0.036).

These results indicate that the participants’ accuracy on the Go/no-go task was significantly higher when identifying the fearful faces compared to happy faces, supporting the “negativity bias” (Carretié et al., 2001; Luo et al., 2010; Zhu and Luo, 2012).

*Reaction time*

A 2 (emotion of the visible face: happy or fearful) × 3 (emotional consistency between visible and invisible faces: consistent, irrelevant, or inconsistent) repeated-measures ANOVA of reaction time revealed that the main effect of “emotion of the visible face” was not significant (*F*(1,17) = 0.482, *p* = 0.497, η_*p*_^2^ = 0.028), and the interaction was not significant (*F*(2,34) = 0.240, *p* = 0.788, η_*p*_^2^ = 0.014). But the main effect of emotional consistency was significant (*F*(2,34) = 5.224, *p* = 0.011, η_*p*_^2^ = 0.235; the right panel of Figure 2). Bonferroni multiple comparisons revealed that the consistent condition was significantly faster than the inconsistent condition (*p* = 0.032), and the irrelevant condition (i.e., the neutral face condition) was marginally significantly faster than the inconsistent condition (*p* = 0.057).

This demonstrates that even if a face on one side of the visual field is not consciously perceived, its emotion (specifically happiness and fear) can strongly affect the emotional discrimination of the emotional face on the other side of the visual field. When the emotions of visible and invisible faces conflict the discrimination of the visible emotional faces takes significantly longer compared to when emotions are consistent. This evidence supports the unconscious processing of facial expressions at the behavioral level, which is consistent with previous studies (Bertini et al., 2013; Tamietto and Gelder, 2008). In addition, the effect of emotional consistency is primarily demonstrated through the interference effect of conflicting invisible facial expressions toward discriminating the emotions of visible faces.

## 3 Experiment 2: unconscious processing of happy faces and its correlation with prosocial tendency and extraversion

### 3.1 Participants

A repeated-measures, within-factors power analysis in G*Power revealed a minimum sample size of 19 to achieve adequate power to detect a medium effect size (parameters: effect size *f*(U) = 0.5, α = 0.05, power = 0.85, number of groups = 1, number of measurements = 4, “as in SPSS” option enabled) (Faul et al., 2009).

Forty-seven sophomores (7 males and 40 females), aged 20 years old, took part in this study. All participants reported normal or corrected-to-normal visual acuity and were naive to the purpose of the experiment. They all gave electronic informed consent in accordance with procedures and protocols approved by the institutional review board of our university and received course bonus points for their participation. To explore the correlation between the unconscious processing of happy faces, prosocial tendency, and extraversion, they finished an online questionnaire measuring prosocial tendency and extraversion personality traits after the experiment.

### 3.2 Materials

#### 3.2.1 Apparatus and display

The experiment was conducted on the laptop of each student at home. The experimental program was written in E-Prime 2.0.

#### 3.2.2 Stimuli

**FIGURE 3:**
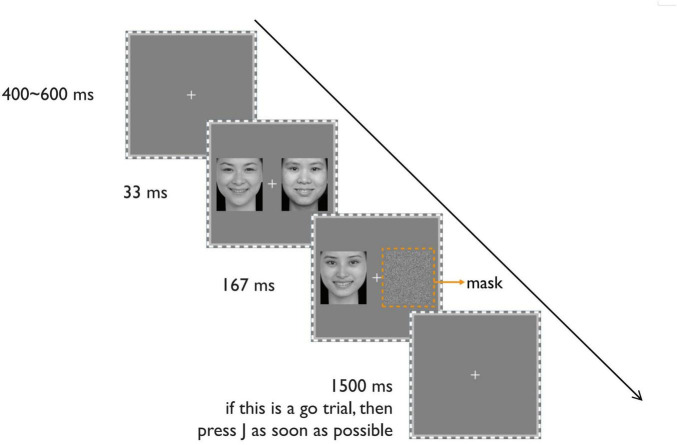
The schematic display of the procedure of a typical trial. Reproduced with permission from Gong et al. (2011).

From the Chinese Emotional Face System (Gong et al., 2011), we selected pictures of happy and neutral faces with straight heads and hairless faces. Eight (4 male and 4 female) happy faces, with open-mouthed broad smiles, and eight (4 male and 4 female) neutral faces were selected. For the pictures used to backwardly mask the happy or neutral faces, a new neutral face was selected and spatially scrambled (see Figure 3). Another four (2 male and 2 female) happy faces and another four (2 male and 2 female) neutral faces were selected for the practice phase.

Finally, we adjusted the average RGB luminance of each face to around 135 through Photoshop software.

#### 3.2.3 Questionnaires

Two measures were used in this study: the revised Chinese edition of Prosocial Tendency Measure (PTM) (Kou et al., 2007) and the extraversion subscale of the revised Chinese edition of the simplified NEO-FFI (Neuroticism Extraversion Openness Five-Factor Inventory) (Yao and Liang, 2010).

The revised PTM consists of 26 questions assessing one’s tendency to help other people during various conditions, which has high reliability and validity (Kou et al., 2007).

The extraversion subscale of the revised Chinese edition of the simplified NEO-FFI (Neuroticism Extraversion Openness Five-Factor Inventory) comprises 12 questions measuring the extraversion personality, which also shows high reliability and validity (Yao and Liang, 2010).

### 3.3 Procedure

As shown in Figure 3, each trial starts with a fixation at the center of the screen for 400–600 ms. Then, on one side of the screen (randomly left or right side), a happy or neutral face appears for 33 ms, followed by a spatially scrambled neutral face for 167 ms as a backward mask. Simultaneously, the other side of the screen displays two successive faces for 33 and 167 ms, respectively. These faces share the same expression (happy or neutral) but have different identities. *The manipulation of the visible side differs from Experiment 1 to make the two visual fields as similar as possible.*

Before the formal experiment, the participants practice until they are familiar with the task. The experimental task, identical to Experiment 1, is the classical Go/no-go task (Tamietto and Gelder, 2008). Participants must press the J key on the keyboard as quickly as possible when they detect the target facial expression (Go response) and not respond when they detect the other non-target expression (No-go response). The target expression for the Go reaction is fixed in each block. The target face is the 167-ms visible (either happy or neutral) face. Each trial has a time limit of 1500 ms. The next trial begins after 1500 ms or a key response.

The formal experiment consists of 2 blocks, each containing 64 trials. The target expression for the Go reaction is fixed within each block. For half the participants, the target expressions for two blocks are happy and then neutral (Happy-Neutral sequence). For the other half, the target expressions for two blocks are neutral and then happy (Neutral-Happy sequence). These two sequences are balanced across participants.

Different from Experiment 1, we ensured all three faces were of the same gender during each trial. Besides, the identities of visible-side faces were completely different from those of the visible-side faces. Therefore, the masked faces were never exposed at a conscious level.

At the end of the experiment, the participants complete an awareness check task. This task follows the same procedure as the formal experiment but consists of only 16 trials. Participants must choose or guess whether the briefly presented face before the mask (that is, the 167-ms scrambled neutral faces) is happy or neutral.

### 3.4 Experimental design

The experiment employs a 2 (emotion of the visible face: happy or neutral) × 2 (emotional consistency between visible and invisible faces: consistent or inconsistent) within-subject design, which is different from Experiment 1. As a result, there are four experimental conditions (Table 1). Each condition has 32 trials—16 Go trials and 16 No-go trials.

**TABLE 1:** All four experimental conditions and their abbreviations.

Abbreviations of experimental conditions	Experimental conditions	Visible face	Invisible face
TT	Neutral consistent	Neutral face	Neutral face
TP	Neutral inconsistent	Neutral face	Happy face
PP	Positive consistent	Happy face	Happy face
PT	Positive inconsistent	Happy face	Neutral face

P, positive; T, neutral. The first letter indicates the emotion of the visible face, while the second letter represents the emotion of the invisible face.

### 3.5 Results and discussions

#### 3.5.1 Exclusion criteria

We calculated the mean and standard deviation for 47 participants on these indices: the overall accuracy and reaction time, and the accuracy of the awareness check task. Unlike Experiment 1, we did not calculate the *d*’ by the signal detection theory method due to the small number of trials in the awareness check task for Experiment 2.

Three participants’ overall accuracy of the Go/no-go task exceeded the mean–2.5SD of all participants. No participants’ accuracy excluded the mean + 2.5SD of all participants in the awareness check task.

Consequently, data from 44 participants were considered valid for analysis. Their mean accuracy of Go trials was 0.93, while the mean false alarm rate of No-go trials was 0.23.

#### 3.5.2 Awareness check results

In the final awareness check task, the mean accuracy of all participants was 0.581, significantly higher than 0.5 (i.e., chance-level accuracy) (*t*(43) = 3.468, *p* ≈ 0.001). This suggests that some participants could recognize the 33-ms masked happy or neutral face.

Therefore, we divided them into two groups using an accuracy threshold of 0.6. Twenty-two participants formed the high-accuracy group, with a mean accuracy of 0.701, significantly higher than 0.5 (*t*(21) = 13.575, *p* < 0.001). The remaining 22 participants formed the low-accuracy group, with a mean accuracy of 0.461, not significantly different from 0.5 (*t*(21) = –1.54, *p* = 0.139). Therefore, the low-accuracy group was named the “unaware group,” while the high-accuracy group was named the “partially aware group.”

#### 3.5.3 Unconscious processing of emotional faces

(1) ***Unaware group***

**FIGURE 4:**
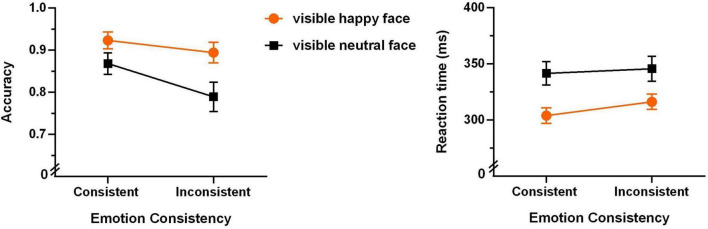
Accuracies **(left panel)** and reaction times **(right panel)** of the Go/no-go task of the unaware group (*N* = 22, the error bars denote ± 1 SEM).

To examine the strictly unconscious effect, we first analyzed the data of the unaware group. The mean accuracies and reaction times for the four experimental conditions are shown in Figure 4.

***Accuracy***

A 2 (emotion of the visible face: happy or neutral) × 2 (emotional consistency between visible and invisible faces: consistent or inconsistent) repeated-measures ANOVA of accuracy revealed that the main effect of emotional consistency was very significant (*F*(1,21) = 15.039, *p* < 0.001, η^2^ = 0.417). As shown in Figure 4, the consistent condition shows significantly higher accuracy than the inconsistent condition. The main effect of “emotion of the visible face” was also very significant (*F*(1,21) = 15.051, *p* < 0.001, η_*p*_^2^ = 0.417), with significantly higher accuracy for happy than neutral faces. The interaction between them was marginally significant (*F*(1,21) = 3.789, *p* = 0.065, η_*p*_^2^ = 0.153).

***Reaction time***

A 2 (emotion of the visible face: happy or neutral) × 2 (emotional consistency between visible and invisible faces: consistent or inconsistent) repeated-measures ANOVA of reaction time only revealed a significant main effect of “emotion of the visible face” (*F*(1,21) = 18.484, *p* < 0.001, η_*p*_^2^ = 0.468). As shown in Figure 4, the happy face condition shows significantly shorter reaction time than the neutral face condition. The main effect of emotional consistency was not significant (*F*(1,21) = 1.192, *p* = 0.154, η_*p*_^2^ = 0.095), and the interaction between them was not significant (*F*(1,21) = 0.522, *p* = 0.478, η_*p*_^2^ = 0.024).

This result indicates that the participants’ reaction time on the Go/no-go task is significantly shorter when identifying the happy face than the neutral face. Along with the results of accuracy, they all support the happy face recognition advantage in extrafoveal vision (Calvo et al., 2010).

**(2) *Partially aware group***

**FIGURE 5:**
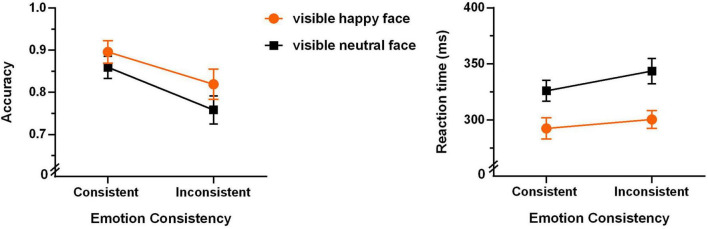
Accuracies **(left panel)** and reaction times **(right panel)** of the Go/no-go task of the partially aware group (*N* = 22, the error bars denote ± 1 SEM).

Furthermore, we analyzed the data of the partially aware group. The mean accuracies and reaction times for the four experimental conditions are shown in Figure 5.

***Accuracy***

A 2 (emotion of the visible face: happy or neutral) × 2 (emotional consistency between visible and invisible faces: consistent or inconsistent) repeated-measures ANOVA of accuracy revealed that the main effect of emotional consistency was very significant (*F*(1,21) = 8.606, *p* = 0.008, η^2^ = 0.291). As shown in Figure 5, the consistent condition shows significantly higher accuracy than the inconsistent condition. The main effect of “emotion of the visible face” was marginally significant (*F*(1,21) = 3.598, *p* = 0.072, η_*p*_^2^ = 0.146), with higher accuracy for happy than neutral faces. The interaction between them was not significant (*F*(1,21) = 1.481, *p* = 0.237, η_*p*_^2^ = 0.066).

***Reaction time***

A 2 (emotion of the visible face: happy or neutral) × 2 (emotional consistency between visible and invisible faces: consistent or inconsistent) repeated-measures ANOVA of the reaction time revealed significant main effect of “emotion of the visible face” (*F*(1,21) = 8.636, *p* = 0.008, η_*p*_^2^ = 0. 291) and marginally significant main effect of “emotional consistency” (*F*(1,21) = 3.686, *p* = 0.069, η_*p*_^2^ = 0.149). As shown in Figure 5, happy faces show significantly shorter reaction time than neutral face, and the consistent condition shows shorter reaction time than the inconsistent condition. The interaction effect was not significant (*F*(1,21) = 1.228, *p* = 0.280, η_*p*_^2^ = 0.055).

Comparing the results of the two groups would demonstrate that the emotional consistency effect is robust regardless of awareness level, primarily reflected in the accuracy index.

#### 3.5.4 Calculation of the indices of unconscious processing of emotional face

The unconscious processing of invisible happy faces was reflected in two aspects:

(1) ***the promotion effect*** of invisible happy expressions on identifying happy faces, which is quantified by ACC_*PP*_ – ACC_*PT*_ and RT_*PT*_ – RT_*PP*_ (see Table 1 for the explanation of the abbreviations). The across-participant mean Promotion Effect can be observed from the slope of the orange lines in Figures 4, 5.

(2) ***the interference effect*** of invisible happy expressions on identifying neutral faces, which is quantified by ACC_*TT*_ – ACC_*TP*_ and RT_*TP*_ – RT_*TT*_. In the same vein, the across-participant mean Interference Effect can be observed from the slope of the black lines in Figures 4, 5.

The larger the above 4 indices, the stronger the unconscious processing of happy faces.

#### 3.5.5 Correlation analysis

After calculating the above indices, Pearson correlation analysis was performed on the above 4 indices and scores of two questionnaires.

*(1)* ***Unaware group***

As shown in Table 2, three significant correlations were found for the unaware group.

**TABLE 2:** Correlations between all the behavioral indices and personalities for the unaware group.

	1	2	3	4	5
1 Prosocial tendency					
2 Extraversion	**−0.039**				
3 Promotion effect (ACC)	0.280	0.006			
4 Interference effect (ACC)	0.488*	**−**0.299	0.089		
5 Promotion effect (RT)	0.406†	0.122	0.577**	0.444*	
6 Interference effect (RT)	0.320	**−**0.145	0.161	0.224	**−**0.018

*p < 0.05,

**p < 0.01,

†0.05 < *p* < 0.1. ACC, accuracy; RT, reaction time.

First, the promotion effect of reaction time (i.e., RT_*PT*_ – RT_*PP*_) and the promotion effect of accuracy (i.e., ACC_*PP*_ – ACC_*PT*_) were very significantly positively correlated [*r*(22) = 0.577, *p* = 0.005, FDR-corrected *p* = 0.005]. In addition, the promotion effect of reaction time (i.e., RT_*PT*_ – RT_*PP*_) and the interference effect of accuracy (i.e., ACC_*TT*_ – ACC_*TP*_) were significantly positively correlated [*r*(22) = 0.444, *p* = 0.039, FDR-corrected *p* = 0.045].

**FIGURE 6:**
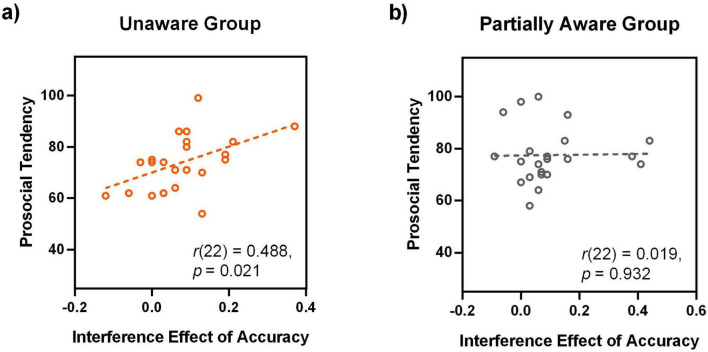
Correlation between prosocial tendency and the processing of happy faces for the unaware **(A)** and partially aware **(B)** groups. Note that the panel **(B)** shows non-significant correlation for the partially aware group.

Second, the prosocial tendency score and the interference effect of accuracy (i.e., ACC_*TT*_ – ACC_*TP*_) were significantly positively correlated [*r*(22) = 0.488, *p* = 0.021, FDR-corrected *p* = 0.023] (Figure 6A). This result indicates that there is a significant correlation between the unconscious processing of happy expressions and the prosocial tendency.

*(2)* ***Partially aware group***

As shown in Table 3, only two significant correlations were found for the partially aware group.

**TABLE 3:** Correlations between all the behavioral indices and personalities for the partially aware group.

	1	2	3	4	5
1 Prosocial tendency					
2 Extraversion	0.540*				
3 Promotion effect (ACC)	0.100	0.234			
4 Interference effect (ACC)	0.019	0.046	0.808**		
5 Promotion effect (RT)	0.216	**−**0.283	0.155	0.150	
6 Interference effect (RT)	**−**0.132	**−**0.232	**−**0.014	0.035	0.406^†^

*p < 0.05,

**p < 0.01,

†0.05 < *p* < 0.1. ACC, accuracy; RT, reaction time.

First, the promotion effect of accuracy (i.e., ACC_*PP*_ – ACC_*PT*_) and the interference effect of accuracy (i.e., ACC_*TT*_ – ACC_*TP*_) were very significantly positively correlated [*r*(22) = 0.808, *p* < 0.001, FDR-corrected *p* < 0.001].

Second, the prosocial tendency score significantly positively correlated with extraversion [*r*(22) = 0.540, *p* = 0.009, FDR-corrected *p* = 0.01], which is quite different from the results of the unaware group (as shown in Table 2) and the overall correlation result ([*r*(44) = 0.215, *p* = 0.161]).

For a direct comparison with the unaware group, we also presented the correlation between the prosocial tendency score and the interference effect of accuracy (i.e., ACC_*TT*_ – ACC_*TP*_), which is not significant at all (Figure 6B).

Taken together, a positive correlation was demonstrated between the individual prosocial tendency and the processing of happy faces, specifically for the unaware group. And the correlation was reflected in the interference effect of accuracy.

## 4 General discussion

Previous studies have shown that the brain can process unconscious (i.e., invisible) emotional faces (Bertini and Làdavas, 2021; Diano et al., 2016; Jiang and He, 2006; Pegna et al., 2005; Qiu et al., 2022; Tamietto and De Gelder, 2010; Wang et al., 2023), which influence subsequent cognition and behaviors (Almeida et al., 2013; Anderson et al., 2012; Gruber et al., 2016; Sagliano et al., 2020; Vetter et al., 2019). Consistent with previous behavioral studies (Bertini et al., 2013; Tamietto and Gelder, 2008), the ANOVA results of the present study (both Experiment 1 and Experiment 2) provide additional behavioral-level evidence for the unconscious processing of emotional faces (specifically happy and fearful faces). Furthermore, Experiment 2 revealed a significant correlation between the behavioral index of unconscious processing of extrafoveal happy faces and one’s prosocial tendency. However, no such correlation was found with extraversion personality

The correlation results of Experiment 2 revealed that the larger the prosocial tendency of an individual, the stronger the interference effect of unconscious happy expressions on identifying neutral faces. Facial expressions, conveying non-verbal social cues like social intentions, play an important role in social communications (Frith, 2009; Tracy et al., 2015). For example, happy expressions convey trust, friendliness, approval, or liking, and fearful expressions indicate vigilance or potential dangers. In Experiment 2, we focused solely on happy faces, omitting fearful faces, for two reasons. First, the unconscious processing of fearful faces has been extensively studied, and prior studies already demonstrated a correlation between the neuroimaging response to subliminal fearful faces and psychopathology-related traits such as negative affectivity (including anxiety, depression, neuroticism, and so on) (Günther et al., 2020; Vizueta et al., 2012). Second, the happy facial expression is the most recognizable facial expression, serving as a positive sign of prosocial intentions even in the most remote cultures (Ekman and Friesen, 1971). Happy faces are identified most accurately and quickly compared to other facial expressions, even in the extrafoveal visual field (Calvo et al., 2010). Moreover, happy faces can attract attention in the dot-probe paradigm, which could not be attributed to low-level factors (Wirth and Wentura, 2020). The correlation results of Experiment 2 strongly supported one of the social communicative functions of happy face perception: receiving prosocial messages from the happy faces and thereby promoting prosocial behavior toward them. This result is in keeping with a previous study where participants were trained to perceive happy rather than angry expressions on an emotionally ambiguous face, leading to a decrease in self-reported state anger and aggressive behaviors of adolescents at high risk of criminal offending and delinquency (Penton-Voak et al., 2013). Therefore, the perception of subtle expressions of happiness might causally promote prosocial tendencies and reduce antisocial behaviors like aggression. Future studies could further examine this possibility, potentially paving the way for unconscious interventions to encourage prosocial behavior and discourage antisocial actions.

Besides, Killgore and Yurgelun-Todd (2007) suggested that empathy and prosocial behaviors might be related to the ability to detect subtle expressions of sadness because sad facial expressions communicate loss and the need for social support. Consequently, further research into sad facial expressions is warranted. Notably, future studies should consider dynamic facial expressions rather than static ones, as dynamic expressions are more effective in inducing unconscious emotional responses than static expressions (Sato et al., 2014).

Some researchers assumed that the results of unconscious processing of faces could be attributed to partial or residual awareness (Kleckner et al., 2018; Kouider et al., 2010). It should be noted that the correlation result of Experiment 2 is restricted to the unaware group, (Figure 6 and Tables 2, 3). Therefore, it demonstrated a dissociation between the conscious and unconscious levels. As Merikle and Daneman (2000) noted, a qualitative difference between unconscious-level and conscious-level results is the most valid indicator of unawareness. Thus, the distinct patterns of correlation results support the notion that the unconscious effect in the present study is restricted to the unconscious level, but not due to partial or residual awareness. Some may argue that a 33-ms presentation is long enough to produce partial awareness. However, it should be noted that the 33-ms face was presented to the extrafoveal vision, and participants could not predict which visual field the conscious face would appear in. These factors made it difficult to detect the 33-ms extrafoveal face. In addition, the extrafoveal presentation of facial expressions more closely resembles everyday life, as we don’t always gaze directly at others’ faces. Our results suggest that in daily life, prosocial individuals are more likely to detect happy facial expressions during extrafoveal vision, even with limited clues.

The current study had several limitations. First, the awareness check procedure was not conducted on a trial-by-trial basis, which might be considered insufficiently rigorous. During some trials, participants’ responses may be influenced by partial awareness. However, if the awareness check is conducted in a trial-wise fashion, the secondary task might interfere with the main task by interrupting transitions from one trial to the next (Cheng et al., 2019; Rabagliati et al., 2018). Secondly, there is a long-lasting debate on how to measure the efficacy of backward masking techniques (Wiens, 2006). To ensure strict unconsciousness of backward-masked stimuli, the experimenters should present forced-choice questions about the emotional valence of each masked stimulus. The present study adhered to this protocol. Some researchers argue that *d*’ = 0 may not be sufficient to guarantee the absence of consciousness. This is because *d*’ = 0 is a null hypothesis lacking statistical power. Additionally, *d*’ = 0 might result from low motivation rather than a true lack of awareness (Merikle and Daneman, 2000; Wiens, 2006). Future research could implement a trial-by-trial awareness check procedure, asking participants to rate their level of awareness. This approach would help study the dose-response relationship between changes in awareness and other variables (Wiens, 2006). Lastly, to achieve a more balanced gender representation, more male participants should be enrolled to bring the male-to-female ratio closer to 1:1. This will help determine whether the correlation effect is consistent across genders.

In summary, few studies have examined the unconscious processing of extrafoveal happy faces. The present study underscores the need for future research into the unconscious processing of extrafoveal happy expression and calls for more investigation into the adaptive functions of unconscious emotional face processing.

## 5 Conclusion

(1) The discrimination of visible emotional faces was modulated by the facial expression of the invisible face in the opposite visual field. Emotionally consistent conditions showed shorter reaction time (Experiment 1) or higher accuracy (Experiment 2) than inconsistent conditions.

(2) The unconscious processing of emotional face is positively correlated with individual prosocial tendency, but not extraversion. These results shed new light on the functional role of the unconscious processing of happy expressions for the first time and support the social-communicative function of facial expressions.

## Data Availability

The original contributions presented in this study are included in this article/Supplementary material, further inquiries can be directed to the corresponding author.
